# Therapeutic Advances for Huntington’s Disease

**DOI:** 10.3390/brainsci10010043

**Published:** 2020-01-12

**Authors:** Ashok Kumar, Vijay Kumar, Kritanjali Singh, Sukesh Kumar, You-Sam Kim, Yun-Mi Lee, Jong-Joo Kim

**Affiliations:** 1 Department of Genetics, Sanjay Gandhi Post-Graduate Institute of Medical Sciences, Lucknow 226014, UP, India; chemistry.ashok83@gmail.com; 2 Department of Biotechnology, Yeungnam University, Gyeongsan, Gyeongbuk 38541, Korea; samsam5332@naver.com (Y.-S.K.); ymlee@yu.ac.kr (Y.-M.L.); 3 Central Research Station, Subharti Medical College, Swami Vivekanand Subharti University, Meerut 250002, India; skritanjali@gmail.com; 4 PG Department of Botany, Nalanda College, Bihar Sharif, Magadh University, Bihar 824234, India; kumarsukesh92@gmail.com

**Keywords:** Huntington’s disease, CAG repeat, mutant huntingtin (mHTT), therapeutics, neurodegeneration

## Abstract

Huntington’s disease (HD) is a progressive neurological disease that is inherited in an autosomal fashion. The cause of disease pathology is an expansion of cytosine-adenine-guanine (CAG) repeats within the huntingtin gene (*HTT*) on chromosome 4 (4p16.3), which codes the huntingtin protein (mHTT). The common symptoms of HD include motor and cognitive impairment of psychiatric functions. Patients exhibit a representative phenotype of involuntary movement (chorea) of limbs, impaired cognition, and severe psychiatric disturbances (mood swings, depression, and personality changes). A variety of symptomatic treatments (which target glutamate and dopamine pathways, caspases, inhibition of aggregation, mitochondrial dysfunction, transcriptional dysregulation, and fetal neural transplants, etc.) are available and some are in the pipeline. Advancement in novel therapeutic approaches include targeting the mutant huntingtin (mHTT) protein and the *HTT* gene. New gene editing techniques will reduce the CAG repeats. More appropriate and readily tractable treatment goals, coupled with advances in analytical tools will help to assess the clinical outcomes of HD treatments. This will not only improve the quality of life and life span of HD patients, but it will also provide a beneficial role in other inherited and neurological disorders. In this review, we aim to discuss current therapeutic research approaches and their possible uses for HD.

## 1. Introduction

Huntington’s disease (HD) is genetically inherited in an autosomal dominant fashion. It is a fatal neurodegenerative disease, caused by an abnormal triplet repeat expansion of CAG (cytosine-adenine-guanine) within the huntingtin (*HTT*) gene on chromosome 4p16.3, causing a mutated huntingtin protein (mHTT) [1,2,3,4,5]. HD is predominantly characterized by adult-onset, progressive motor dysfunction, cognitive impairment and psychiatric symptoms (depression, anxiety, obsessive-compulsive disorder, and psychosis). Chorea, incoordination, and rigidity are common motor symptoms due to neurotoxicity of mHTT, leading to brain atrophy of the striatum, thalamus, cerebellum, brain stem and cortex [6,7,8,9]. Clinically, HD includes juvenile HD (onset less than 21 years, and marked clinical symptoms), and late-onset HD (after the age of 60 years) [10,11,12]. Alcohol, drug, and tobacco abuse were associated with earlier onset of HD, and hasten motor onset in women. These abuses have more significant associations in females than in males [13,14]. Children with CAG repeats ≥39, had significantly lower measures of head circumference, weight, and body mass index [15,16,17]. Disrupted sleep, tics, pain, itching, and psychosis are the common symptoms of juvenile HD [18].

Presently, there is no remedy for HD, and the disease progresses manifests with a presumed continuation of 15–20 years after the appearance of the first symptom [12,19]. The identification of novel biomarkers involves the development of new treatment strategies. The current therapy is palliative and does not change the course of the disease. Tetrabenazine (TBZ; Xenazine™) was approved for the remedy of chorea in HD by the U.S. food and drug administration (FDA). Additionally, the deuterated version of TBZ, deutetrabenazine (AUSTEDO™), has an improved pharmacokinetic profile and was recently approved by the FDA for the treatment of Huntington chorea. In the last review [20], we discussed different promising agents in the treatment of HD, and their phases under clinical trial. Here we describe updates related to these promising agents which will cure HD.

## 2. Pathogenesis of the HD

**Figure 1:**
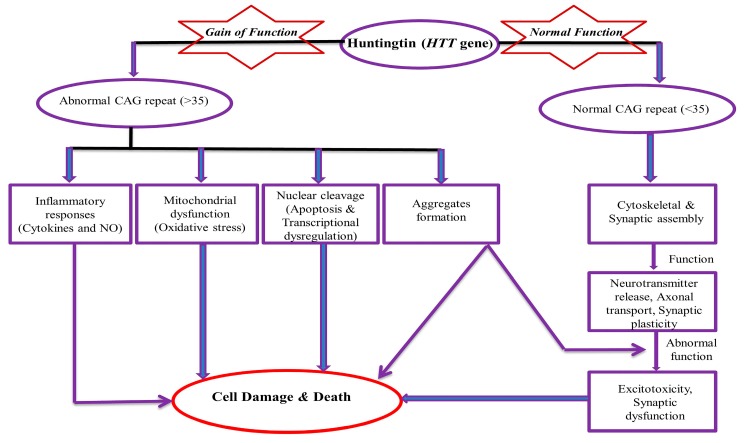
Mechanism of Toxicity of Huntingtin (*HTT*) gene. NO (Nitric Oxide), CAG (cytosine-adenine-guanine).

HD is a monogenic disease with prevalence of about 1 in 7,500 individuals in the general population [21,22]. The normal allele has less than 27 CAG repeats and intermediate alleles have 27–35 repeats. CAG repeats of 36–39 will develop HD with less penetrance. Individuals who have 40 or more CAG repeats will develop HD with full penetrance. It is also reported that the higher the CAG expansion, the earlier the onset and the greater the disease severity [12,23]. Kremer et al. reported the largest expansion of 121 trinucleotides [24]. CAG codon encodes glutamine α-amino acid (symbol Gln or Q). Glutamine (C_5_H_10_N_2_O_3_) is synthesized from glutamate and ammonia by the enzyme glutamine synthetase. It is mainly produced in muscle, the lungs, and the brain and acts as a precursor to the neurotransmitter glutamate [25]. CAG has glutamine amino acids within the *HTT* gene and it is not toxic in itself. However, the polyglutamine expansion involves the formation of aggregate and ultimately becomes toxic. It is the principal factor for the manifestation of HD because aggregates are never a remarkable feature in the brain of normal subjects [26,27]. Aggregate formations are accountable for secondary problems, like inflammatory responses (altered cytokine and nitric oxide level), mitochondrial dysfunction (imbalanced level of free radicals and oxidative stress markers), nuclear cleavage, apoptosis, excitotoxicity, transcriptional altered regulation, and lastly, are responsible for the altered neuropathological feature (cause of cell death/damage) (Figure 1). Approximately 70% of the variation of the disease is due to expanded CAG repeats, while 13% of the variation is due to polymorphisms in the GRIK2 gene [28]. These depict the importance of secondary factors that affect disease onset, its severity, and possible output.

## 3. Therapeutic Update

**Figure 2:**
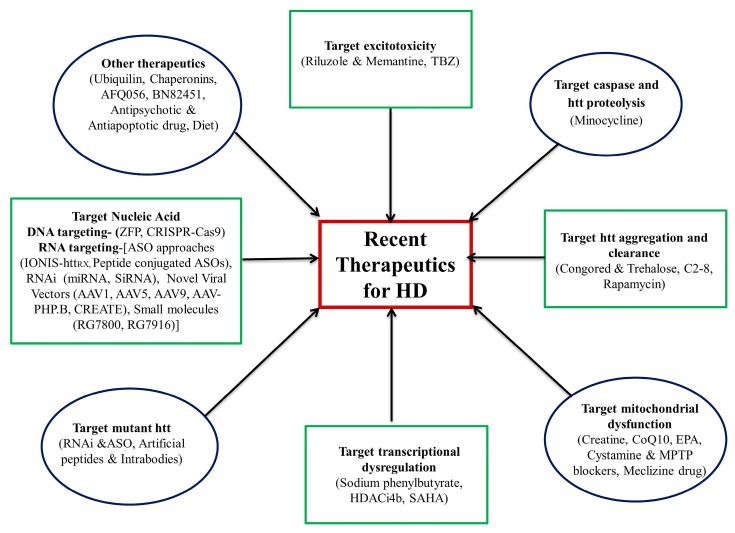
Recent advancement in the therapeutics for Huntington’s disease. TBZ (Tetrabenazine); EPA (eicosapentaenoic acid); MPTP (1-methyl-4-phenyl-1,2,3,6-tetrahydropyridine); SAHA (suberoylanilide hydroxamic acid); HDACi4b (histone deacetylase inhibitors); RNAi (RNA interference); ASO (antisense oligonucleotide); ZFP (zinc finger protein); CRISPR-Cas9 (clustered regularly interspaced short palindromic repeats); miRNA (micro RNA); siRNA (small interfering RNA).

Currently, many drugs are under clinical trial. In the following subsection, we discuss their therapeutic status and their potential role in treatment. These drugs are summarized in Table 1 and Figure 2.

### 3.1. Drugs against Excitotoxicity

#### 3.1.1. Riluzole and Memantine Drug

Riluzole is a glutamate inhibitor that reduces abnormal movement in amyotrophic lateral sclerosis (ALS) patients [29,30]. In a double-blinded trial, riluzole did not decrease symptoms of HD, nor was it neuroprotective [31].

Memantine is an antagonist of extrasynaptic N-methyl-D-aspartate (NMDA) receptors and is used for the treatment of moderate-severe dementia in Alzheimer’s disease (AD). It diminishes striatal cell death, hinders disease progression and improves cognitive function related to HD [32,33]. The combination of memantine and risperidone prevented the expected progression of motor symptoms, cognitive decline, and psychosis over a 6-month study period [34]. However, memantine dosing may be critical, as rodents on low-dose memantine had decreased pathology, while a high-dose of memantine worsened rodent outcomes and possibly promoted cell death [35,36,37].

#### 3.1.2. Tetrabenazine (TBZ) and Deutetrabenazine 

TBZ inhibits the dopamine pathway by inhibiting vesicular monoamine transporter (VMAT) type 2 and consequently decreases available dopamine in the synapse and its interaction with postsynaptic dopamine receptors [38,39,40]. Deutetrabenazine contains a deuterium atom and is a novel inhibitor of VMAT2. In indirect treatment comparison studies, deutetrabenazine was found to have a favorable tolerability profile compared to tetrabenazine [41]. In mouse models, TBZ ameliorated chorea and other motor symptoms, and reduced striatal neuronal cell loss [38].

### 3.2. Targeting Caspase Activities and Huntingtin Proteolysis

#### Minocycline

Minocycline is a tetracycline analog and can cross the blood–brain barrier (BBB) and inhibits the expression of caspase-3 and caspase-1 [42,43]. Treatment with minocycline proved to be neuroprotective, and to improve the disease phenotype [30,42,44]. A human trial study observed motor (unified HD rating scale (UHDRS)), and cognitive (mini-mental state examination (MMSE)) improvement in 14 HD patients who took 100 mg of minocycline for 6 months [43]. This study was continued for another 18 months, and it was found that MMSE, TMS, total functional capacity (TFC) and independence scale were all stabilized after treatment, reducing the expected decline in these measures. There was also a decrease in psychiatric symptoms at 24 months, which was not apparent after 6 months of treatment [44]. In a pilot study, Thomas et al. found improvement in MMSE, UHDRS, and abnormal involuntary movements scale (AIMS), in 30 patients with HD who were given minocycline for 6 months [45].

### 3.3. Targeting HTT Aggregation and Clearance

#### 3.3.1. Congo Red and Trehalose

Congo red dye binds preferably to β-sheets with amyloid fibrils. When injected into HD mice, it preserved normal protein synthesis and degradation, and improved motor functions. This dye promotes clearance of expanded polyQ repeats and inhibits polyglutamine oligomer formation through the disruption of preformed oligomers. Congo red dye also prevented ATP depletion and caspase activation [46,47,48].

Trehalose disaccharide inhibited the formation of nuclear inclusions, improved altered motor function and was associated with a high rate of survival in R6/2 mice without causing harmful side effects [49,50,51] (Table 1 and Figure 2).

#### 3.3.2. Compound C2–8

Compound C2–8 inhibits polyglutamine aggregates in brain slices and cell cultures. It has improved motor function, decreased the amount of neuronal atrophy, and decreased the size of the mHTT aggregates in R6/2 mice [52,53]. There is no ongoing human trial using this compound currently listed on clinicaltrials.gov.

#### 3.3.3. Rapamycin

mTOR is a protein kinase that phosphorylates many proteins and plays a key role in various cellular functions (like autophagy and transcription). mTOR interacts with mHTT and localizes to these polyglutamine aggregates, and thus sequestration of mTOR reduces the activity of mTOR, resulting in a decrease in autophagy and a decrease in the clearance of mHTT. mTOR phosphorylates S6K1 (a key regulator of cell volume), therefore mHTT-related impairment of mTOR may account for the brain atrophy in HD. Rapamycin (which inhibits mTOR and consequently induces autophagy) decreased mHTT aggregates and improved neuronal survival in the drosophila HD model. Rapamycin also improved motor performance and decreased striatal neuropathology in mouse models of HD [54,55,56].

### 3.4. Targeting Mitochondrial Dysfunction

#### 3.4.1. Creatine

Creatine (with antioxidant properties) reduced serum levels of 8-hydroxy-2′-deoxyguanosine (8-OH-2′-dG) in HD patients [85,86] and is safe and tolerable at a dose of 15 g twice daily [87]. In a trial study (with HD patients), receiving a dose of 8 g/day of creatine was secure and well-tolerated but produced no marked change on the UHDRS scale [86]. A randomized double-blind study trial measuring TFC (up to 40 g daily) was terminated early due to futility criteria being reached. The use of creatine fails to delay functional decline in an early manifestation of HD [57]. In another controlled study, creatine (5 g/day; 1 year) treatment resulted in better muscle function capacity in patients with neuromuscular disease but did not show improvements in neuromuscular function and the cognitive status of stage I–III HD patients [88].

#### 3.4.2. Coenzyme Q10

Coenzyme Q10 cofactor is involved in ATP production in the electron transport chain (ETC) of mitochondria, and its supplementation in HD patients may improve mitochondrial function [89]. It was neuroprotective in R6/2 mice, delaying motor deficit, atrophy, and inclusion, and extending survival [90,91]. In a phase III randomized clinical trial, coenzyme Q10 was not effective and the trial was stopped as the futility criteria were reached (http://hdsa.org/wp-content/uploads/2015/01) [58].

#### 3.4.3. Eicosapentaenoic Acid (EPA)

Ethyl-EPA is a derivative of the n-3 polyunsaturated fatty acid EPA, which binds to the peroxisome proliferator-activated receptor of mitochondria [92]. Ethyl-EPA improves the neuronal function by inhibiting caspase and reducing mitochondrial damage by reducing the activity of the c-Jun N-terminal kinase (JNK) pathway [93,94]. Treatment with ethyl-EPA (2 g/day) showed a stable/improved motor function. However, intent-to-treat analysis showed no significant change between ethyl-EPA and placebo for total motor score 4 (TMS–4) subscale in HD patients (stage III) [95]. Patients with fewer CAG repeats showed significant improvement in TMS-4. 

In a phase III, double-blind randomized control trial, ethyl-EPA did not show improvement in TMS, cognition or global impression over 6 months. After 6 months, all participants (both those in the treatment and placebo group) were given ethyl-EPA. Those in the original treatment group showed a better motor function (indicated by TMS scores). This suggests that ethyl-EPA needs a longer period before improvement can be observed which might possibly reflect a disease modification [96]. In a recent study, no significant improvement of the treatment group over placebo group was found in measures of TMS or UHDRS scores [59].

#### 3.4.4. Cystamine and MPTP (1-methyl-4-phenyl-1,2,3,6-tetrahydropyridine) Blockers

Both cystamine and MPTP increase the survival effects of HD cells and inhibit oxidative damage [60].

#### 3.4.5. Meclizine

Meclizine, an antihistamine drug, inhibits oxidative metabolism and apoptosis, and is neuroprotective in drosophila model. Energy metabolism deficits and neuronal degeneration are hallmarks of HD, so treatment with meclizine is a potential strategy, especially since it crosses the BBB [20,61] (Table 1 and Figure 2). There is no human clinical trial for this drug listed in clinicaltrials.gov.

### 3.5. Targeting Transcriptional Dysregulation

#### 3.5.1. Sodium Phenylbutyrate

Administration of sodium phenylbutyrate (an HDAC inhibitor) to N171-82Q symptomatic mice showed less brain atrophy and extended survival rates. Further, it increased and decreased histone acetylation and methylation, respectively, in the rodent brain. It also downregulated caspases involved in apoptosis [62]. A dose-response study highlighted that sodium phenylbutyrate was safe, secure, effective and well-tolerable in HD patients [97].

#### 3.5.2. HDACi4b

HDACi4b (a pimelic diphenylamide HDAC inhibitor) improves motor impairment as well as decreases neurodegeneration in mouse models of HD. Oral administration of HDACi4b to mice showed improvement in these motor deficits. These mice also showed less striatal atrophy and brain-size reduction. HDACi4b reversed hypoacetylation of the H3 histone subunit that occurs in the presence of mHTT, and mRNA expression was returned to normal levels [63].

#### 3.5.3. Suberoylanilide Hydroxamic Acid (SAHA)

Histone acetylation in the brain is increased by SAHA (by inhibiting HDAC) which improves motor impairments in transgenic R6/2 mice. SAHA can be taken orally because it crosses the BBB, however, this has not been tested in humans [64].

#### 3.5.4. Mithramycin and Chromomycin

Treatments with mithramycin and chromomycin (anthracyclin derivatives) promote epigenetic histone modifications in cultured R6/2 and N171-82Q transgenic cell lines, providing a basis for clinical trials for HD [65,98,99].

### 3.6. Agents Targeting Mutant Huntingtin

#### 3.6.1. RNA Interference (RNAi) and Antisense Oligonucleotide (ASO)

ASO and RNAi execute their knockdown function by allele and nonallele-selective manners [100,101,102]. As an example, modified ASO (peptide nucleic acid i.e., PNA) enables the selective recognition of the mutant allele and selective inhibition of mHTT expression in human fibroblasts [103]. RNAi reduced neuropathology, improved motor behavior and extended viability in HD [102,104,105].

#### 3.6.2. Intrabodies and Artificial Peptides

In transgenic mouse models of HD like R6/2, N171-82Q, YAC128, and BACHD, treatment with intrabody gene therapy improved body weight, motor function, cognitive, and neuropathological manifestation [66,106].

### 3.7. Nucleic Acid-Targeting Therapies

#### 3.7.1. Therapies Targeting DNA

Currently, zinc finger proteins (ZFPs) and CRISPR-Cas9 (clustered regularly interspaced short palindromic repeats-CRISPR-associated system) are under investigation.

##### ZFPs

ZFPs are one of the most abundant protein groups and have various functions, including regulation of DNA, RNA, and protein function. They can bind to specific sequences of DNA and can be used as therapeutic compounds. ZFPs reduce mHTT expression without affecting the expression of other genes/wild-type *HTT* [67,102,107].

##### CRISPR-Cas9

CRISPR-Cas9 is involved in viral defense mechanisms of bacteria which recognize and destroy foreign DNA. CRISPR-Cas9 is involved in the excision of CAG repeats to make harmless alleles and silence the mHTT expression by insertion of stop codon/missense mutations [68,108,109,110]. In HD140Q-knockin mice, it was demonstrated that CRISPR-Cas9 can be used to reduce mHTT and improve motor function, but not to increase the lifespan of these mice [69]. Ekman et al. showed that CRISPR-Cas9 can be used for mHTT editing, which can extend survival and improve motor function in the R6 mice following intrastriatal delivery [111].

#### 3.7.2. RNA Targeting Therapies

The four major methods to inhibit the function of mHTT mRNA are: ASOs, RNAi compounds, novel viral vectors, and small-molecule splicing modulators.

##### ASO Approaches

ASO are single-stranded DNA (ssDNA) molecules that primarily bind to a specific sequence on RNA and regulate post-transcriptional gene expression [112]. The ssDNA diffuses well in the CNS and is taken up by neurons. Therefore, the injection of ASOs into the cerebrospinal fluid (CSF) results in ubiquitous delivery of drugs and suppresses the production of mHTT [70] (Table 1). However, ASO delivery has some side effects, like thrombocytopenia which was observed in some human trials of ASOs [113]. ASO can ameliorate transcriptional dysregulation and reduce the level of mHTT and improve behavior in the YAC128, YAC18, and BACHD mouse models of HD [114,115].

IONIS-HTTRx is an important ASO. It has 12–25 nucleotides and transforms phosphodiester linkages to phosphorothioate. IONIS-HTTRx caused a remarkable reduction in *HTT* mRNA and protein expression [71]. The injection of ASOs (conjugated with peptides), produced wide CNS distribution and longer life span in the spinal muscular atrophy (SMA) mouse model [116]. In a recent study of phase I–IIa trial, HTTRx lessened the concentration of mutant *HTT* in CSF of HD patients. Therefore, ASO compounds not only suppress the expression of *HTT* mRNA and the huntingtin protein in CNS, but also in CSF [117].

##### RNAi Approaches

RNA interference is a gene-silencing process that uses short interfering RNA (siRNA), short hairpin RNA (shRNA), bi-functional shRNA and microRNA (miRNA). The combination of neural progenitor stem cell therapy and RNAi therapy can ameliorate symptoms in mouse models of HD [118]. In the animal models (R6/1, R62, N171-82Q, RAT AAV-HD70^d^) of HD, siRNA, shRNA, and miRNA treatments have been used to reduce neuropathology and improve motor function [6,72,104]. AMT-130 (adeno-associated virus vector) contains an artificial miRNA which produces a huntingtin-lowering molecule. Side effects include peripheral neuropathy observed in clinical trials of siRNA. RNAi has been tested in rodents and its delivery system has been tested in nonhuman primates [119].

##### Small Molecule Approach

Small molecules like RG7800 showed ocular complications in the Δ7 mouse model of spinal muscular atrophy (SMA) [120], while the phase I trial of the molecule RG7916 (risdiplam) was recently completed (NCT02633709). RG7800 and RG7916 are splicing modifiers, which change the way the pre-mRNA is spliced so that it contains all the information necessary to make a functional protein. They promote the production of a full-length and functional protein from the gene. RG7800 increases the survival motor neuron (SMN) protein level by modifying the splicing of the SMN2 mRNA. RG7800 is shown to promote the inclusion of exon 7 in SMN2 mRNA, generating full-length mRNA, using fibroblasts from an SMA type I patient. In the SMA mouse model, the treatment of RG7800 showed a dose-dependent increase in SMN protein levels [121]. Oral administration of RG7800 in SMA patients increased the functional SMN protein level up to two-fold from baseline [122]. New work is now underway to identify these molecules and their possible role in the lowering of mutant *HTT* gene and protein expression [102,123]. 

### 3.8. Other Therapeutics Advancements

#### 3.8.1. Ubiquilin

Ubiquilin overexpression in R6/2 mice decreased aggregation in the hippocampus and cortex and increased lifespan. However, its overexpression did not improve motor symptoms, and did not change the amount of aggregates in the striatum [75,76].

#### 3.8.2. Chaperonins

TRiC (CCT1-CCT8 subunit) is an example of chaperonin which is involved in the folding of about 9–15% of the normal proteins [77] and inhibits aggregation of mHTT [78]. It reduced the number of inclusions, fibrillar oligomers, and insoluble mHTT fragments.

#### 3.8.3. AFQ056

AFQ056 did not improve chorea in a randomized double-blind clinical trial [79].

#### 3.8.4. BN82451

BN82451 inhibits cyclooxygenases and provides antioxidant, anti-inflammatory and neuroprotective effects [80]. It also improved motor function and survival, decreased brain atrophy, neuronal atrophy, and neuronal mHTT inclusions in the R6/2 mice [81]. Recently, a phase II clinical trial has been completed in male HD patients. As per clinicaltrials.gov (NCT02231580), no results have been published.

#### 3.8.5. Antipsychotic Drugs

Antipsychotic drugs are used to treat chorea associated symptoms because they block or modulate dopamine receptors. Many antipsychotic drugs (especially typical antipsychotics) produce motor dysfunction resembling Parkinson’s disease (PD). Currently, a phase III trial comparing TBZ with olanzapine and tiapridal is under evaluation [39].

#### 3.8.6. Antiapoptotic Drugs

Caspase cleavage (mainly caspase-3 and -6) occur in mHTT [83]. Mutating the caspase cleavage sites on mHTT leads to neuroprotection and prevents neurodegeneration in yeast artificial chromosome (YAC) mice that express mHTT. Caspase-3 and -6 resistant mice did not develop HD neurodegeneration, indicating that cleavage at these caspase sites plays an important role in neurodegeneration of HD [82,123,124].

#### 3.8.7. Diet

Various studies indicate that a Mediterranean-type diet may delay the onset of other neurodegenerative diseases, like AD, PD, dementia and cognitive impairment [125]. Recently a study highlighted that a Mediterranean-type diet affects the time to HD phenoconversion. In fact, eating high amounts of dairy products was associated with an increased risk of phenoconversion. This may be due to a lower level of urate, which leads to a faster progression and manifestation of HD. These types of diet-related studies need further investigation [84,107,126]. Intermittent fasting promotes autophagy and cleared the mHTT [127]. 

### 3.9. Some Promising Clinical Trials

#### 3.9.1. Cysteamine (CYST)

Cysteamine controls oxidation levels through increasing concentration of glutathione, activation of protein catabolism through the hindrance of transglutaminase and induction of heat shock proteins (HSPs) effects [128]. Inhibition of transglutaminase is the putative mode of action and is observed in R6/2 and zQ175 mouse models of HD [129]. The Cysteamine-HD phase II/III trials indicated a delay in the release of cysteamine in HD patients.

#### 3.9.2. Pridopidine

Pridopidine is a modulator of the dopamine 2 receptor [130] and activates the sigma-1 receptor [131]. In the most recent trials of pridopidine, no improvement in motor symptoms (unified Huntington’s Disease rating scale - total motor score (UHDRS-TMS)) was observed with placebo [132,133,134]. High level of pridopidine is found in brain-derived neurotrophic factor (BDNF) and diminishes mHTT aggregate size and improved motor performance in R6/2 mice [135,136]. In an early-stage HD, improvement in the total motor score (TMS) was observed in treated patients [136,137].

#### 3.9.3. Triheptanoin

Triheptanoin is a triglyceride that reverses the metabolic defects in HD by supplying substrates to the Krebs cycle [138]. Recently, a phase II study using triheptanoin was conducted in the early phase of HD patients (listed at clincialtrials.gov under #NCT02453061). 

#### 3.9.4. Latrepirdine (Dimebon)

Latrepirdine stabilize and enhance mitochondrial membranes and functions. In a short duration trial, latrepirdine promoted cognitive improvement (MMSE) in mild to moderate HD patients [11,139]. At the time of writing this, a total of 13 clinical trials using latrepirdine have been reported on clinicaltrials.gov (NCT00497159, NCT01085266, NCT00920946, NCT00387270, NCT00988624, NCT00827034, NCT00990613, NCT00824590, NCT00931073, NCT00831506, NCT00788047, NCT00825084).

#### 3.9.5. Amantadine

Amantadine is a weak NMDA receptor blocker [140] and increases dopamine release [141]. Amantadine reduces dyskinesias in HD, without provoking parkinsonism [142]. 

#### 3.9.6. Lamotrigine

Lamotrigine is an antiepileptic drug and decreases glutamate release by blocking voltage-gated sodium channels [143,144]. It reduces motor symptoms and elevates mood in HD patients [145]. 

#### 3.9.7. Selisistat

Selisistat is a selective SirT1 inhibitor which removes acetyl groups on proteins, including mHTT. Therefore, blocking the deacetylation of mHTT should activate clearance. In early-stage HD patients, selisistat improved TMS, but not in most measures of cognition, mood, and functionality [146,147].

#### 3.9.8. Tauroursodeoxycholic Acid/Ursodiol

Tauroursodeoxycholic acid (TUDCA) is a bile acid and has antiapoptotic properties in a mouse model of HD. Mice given TUDCA showed less striatal atrophy, apoptosis, and reduced locomotor and sensory-motor defects [148]. A commercially available uroursodeoxycholic acid precursor, ursodiol, has been examined in a phase I trial, but to date, not reported.

#### 3.9.9. Laquinimod

Laquinimod reduces the expression of Bax, responsible for the release of cytochrome C from mitochondria and activation of caspases, causing apoptosis and production of toxic mHTT fragments. This drug improves motor function and reduces depressive behaviors in mice. It is recently undergoing a phase II clinical trial in human HD patients [149]. Laquinimod ameliorates myelination deficiency and behavioral deficits in the YAC128 mouse model of HD [150,151].

#### 3.9.10. Kynurenine Inhibitors

The enzyme indoleamine 2,3 dioxygenase (IDO1) catalyzes the conversion of tryptophan into kynurenine. Kynurenine is then metabolized into 3-hydroxykynurenine (3-HK) and quinolinic acid, both of which are neurotoxic and are increased in HD. In contrast, kynurenine is also metabolized into kynurenic acid, which is neuroprotective. In HD, an imbalance exists between the neurotoxic products and neuroprotective products and targeting the rate-limiting step of IDO1 could effectively shift the balance toward neuroprotective [152]. Kynurenine 3-monooxygenase is the enzyme that catalyzes the conversion of kynurenine into 3-HK. Treating microglial cells from R6/2 mice with a kynurenine 3-monooxygenase inhibitor (Ro 61–8048) showed dramatically reduced 3-HK levels compared to the vector containing cells [153]. 

## 4. Conclusions and Future perspectives

The current therapeutic investigations of HD mainly focus on excitotoxicity, dopamine pathway, caspase inhibitors, mHTT aggregation, mitochondrial dysfunction, transcriptional dysregulation, and diet. The application of robust molecular imaging and digital biomarkers may provide a valuable therapeutic boost to the design of clinical trials. Additionally, the increased openness of regulatory agencies for effectiveness will also promote the development of clinical trials. The advancement of modern technologies, and the availability of various promising agents/molecules enable the development of therapies which will further improve the quality of research and outcomes in HD patients. The most promising drugs are those that target the production of mHTT protein and block its actions.

## Figures and Tables

**Table 1:** Recent status of Huntington’s disease (HD) drug therapy.

Drug/Reagent	Primary Target (Mechanism of Action)	Status and Principal Result	Ref.
**(1) Drugs against excitotoxicity**			
Riluzole	Glutamate release inhibitor	Does not show efficacy in human trails	[31]
Memantine	N-methyl-D-aspartate (NMDA) receptor inhibitor	Demonstrated efficacy in human trial	[32,33]
Tetrabenazine (TBZ)	Dopamine pathway (Vesicular monoamine transporter 2 inhibitor)	Approved by food and drug administration (FDA) for treatment of chorea in HD	[38,39,40]
**(2) Targeting Caspase and huntingtin (HTT) proteolysis**			
Minocycline	Caspase-dependent and independent neurodegenerative pathway	Inhibits caspase-1 and -3 mRNA upregulation, and decreases inducible nitric oxide synthetase activity	[42,44,45]
**(3) Targeting *HTT* aggregation and clearance**			
Congo red and Trehalose	Aggregation	Showed efficacy in a rodent model	[46,49]
Compound C2–8	Aggregation	Showed efficacy in a rodent model	[53]
Rapamycin	Aggregation mammalian target of rapamycin (mTOR) inhibitor	Showed efficacy in a rodent model	[54,55]
**(4) Targeting mitochondrial dysfunction**			
Creatine	Mitochondrial dysfunction	Attained futility in human trial	[57]
CoQ10	Mitochondrial dysfunction	Attained futility in human trial	[58]
Eicosapentaenoic acid (EPA)	Mitochondria dysfunction	A mixed scenario of positive and negative trial	[59]
Cystamine and mitochondrial permeability transition pore blockers	Mitochondrial dysfunction	Showed efficacy in a rodent model	[60]
Meclizine drug	Mitochondrial dysfunction	Showed efficacy in the fly model	[61]
**(5) Targeting transcriptional dysregulation**			
Sodium phenylbutyrate	Transcriptional deregulation histone deacetylase inhibitor	Showed efficacy in a rodent model	[62]
HDACi4b (a pimelic diphenylamide HDAC inhibitor)	Transcriptional deregulation histone deacetylase inhibitor	Showed efficacy in a rodent model	[63]
Suberoylanilide hydroxamic acid	Transcriptional deregulation histone deacetylase inhibitor	Showed efficacy in a rodent model	[64]
Mithramycin and chromomycin	Transcriptional deregulation G-C-rich DNA binding antibiotic	Showed efficacy in a rodent model	[65]
**(6) Targetting mutant huntingtin (mHTT)**			
RNA interference and antisense oligonucleotide (ASO)	Blocks transcription of mHTT	Showed efficacy in a rodent model	[6]
Artificial peptides and intrabodies	Targeting proline-rich domain of *HTT*	Showed efficacy in a rodent model	[66]
**(7) Therapies targeting nucleic acid**			
Zinc finger protein	Reduced mutant protein expression	Showed efficacy in a rodent model	[67]
CRISPR-Cas9	Excision of CAG repeat and, reduction of mutant *HTT*	Showed efficacy in a rodent model	[68,69]
ASO approach (IONIS-HTTRX, Peptide conjugated ASOs)	Reduction in *HTT* mRNA and protein	Showed efficacy in a rodent model	[70,71]
RNAi approach (siRNA, shRNA, and miRNA)	Improves motor and neuropathological abnormalities, silencing of *HTT*	Showed efficacy in a rodent model	[6,72]
Novel Viral Vectors (AAV1, AAV5, AAV9, AAV-PHP.B, CREATE)	Widespread transduction of cells	Showed efficacy in primate and rodent models	[73,74]
**(8) Other therapeutics**			
Ubiquilin	Reduces mHTT aggregation	Showed efficacy in a rodent model	[75,76]
miRNA	Silence *HTT*	Testing in rodent and nonhuman primates	[6,72]
Chaperonins	Decrease mHTT aggregation	Showed efficacy in a rodent model	[77,78]
AFQ056	The antagonist for glutamate receptor 5	Showed no improvement in chorea in a clinical trial	[79]
BN82451	Decreases glutamate release by blocking Na+ channels	Showed efficacy in a rodent model	[80,81]
Antipsychotic drug	Block or modulate dopamine receptors	Under phase III trial	-
Antiapoptotic drug	Cleave mHTT	Effective in mice model	[82,83]
Diet	Delay onset of disease	Effective result but requires further evaluation	[84]

## Abbreviations

Abnormal involuntary movements scale	AIMS
Alzheimer’s disease	AD
Antisense Oligonucleotide	ASO
Blood-brain barrier	BBB
Brain-derived neurotrophic factor	BDNF
Clustered regularly interspaced short palindromic repeats-CRISPR-associated system	CRISPR-Cas9
Cerebrospinal fluid	CSF
Cytosine-adenine-guanine	CAG
Eicosapentaenoic acid	EPA
Electron transport chain	ETC
Food and Drug Administration	FDA
Huntington disease	HD
Huntingtin gene	*HTT*
micro RNA	miRNA
Mini-mental state examination	MMSE
Mutant huntingtin protein	mHTT
Mammalian target of rapamycin	mTOR
1-methyl-4-phenyl-1,2,3,6-tetrahydropyridine	MPTP
N-methyl-D-aspartate	NMDA
Parkinson’s disease	PD
RNA Interference	RNAi
small interfering RNA	siRNA
Spinal muscular atrophy	SMA
Suberoylanilide hydroxamic acid	SAHA
Tauroursodeoxycholic acid	TUDCA
Tetrabenazine	TBZ
Total functional capacity	TFC
Total motor score	TMS
Unified HD rating scale	UHDRS
Vesicular monoamine transporter 2	VMAT2
Zinc finger proteins	ZFPs
